# The contribution of TMC1 to adaptation of mechanoelectrical transduction channels in cochlear outer hair cells

**DOI:** 10.1113/JP278799

**Published:** 2019-11-12

**Authors:** Adam C. Goldring, Maryline Beurg, Robert Fettiplace

**Affiliations:** ^1^ Department of Neuroscience University of Wisconsin School of Medicine and Public Health Madison WI USA

**Keywords:** adaptation, deafness, hair cells, mechanotransducer channel, transmembrane channel‐like protein

## Abstract

**Key points:**

- Hair cell mechanoelectrical transducer channels are opened by deflections of the hair bundle about a resting position set by incompletely understood adaptation mechanisms.
- We used three characteristics to define adaptation in hair cell mutants of transmembrane channel‐like proteins, TMC1 and TMC2, which are considered to be channel constituents.
- The results obtained demonstrate that the three characteristics are not equivalent, and raise doubts about simple models in which intracellular Ca^2+^ regulates adaptation.
- Adaptation is faster and more effective in TMC1‐containing than in TMC2‐containing transducer channels. This result ties adaptation to the channel complex, and suggests that TMC1 is a better isoform for use in cochlear hair cells.
- We describe a TMC1 point mutation, D569N, that reduces the resting open probability and Ca^2+^ permeability of the transducer channels, comprising properties that may contribute to the deafness phenotype.

**Abstract:**

Recordings of mechanoelectrical transducer (MET) currents in cochlear hair cells were made in mice with mutations of transmembrane channel‐like (TMC) protein to examine the effects on fast transducer adaptation. Adaptation was faster and more complete in *Tmc2^–/–^* than in *Tmc1^–/–^*, although this disparity was not explained by differences in Ca^2+^ permeability or Ca^2+^ influx between the two isoforms, with TMC2 having the larger permeability. We made a mouse mutation, *Tmc1* p.D569N, homologous to a human DFNA36 deafness mutation, which also had MET channels with lower Ca^2+^‐permeability but showed better fast adaptation than wild‐type *Tmc1^+/+^* channels. Consistent with the more effective adaptation in *Tmc1* p.D569N, the resting probability of MET channel opening was smaller. The three TMC variants studied have comparable single‐channel conductances, although the lack of correlation between channel Ca^2+^ permeability and adaptation opposes the hypothesis that adaptation is controlled simply by Ca^2+^ influx through the channels. During the first postnatal week of mouse development, the MET currents amplitude grew, and transducer adaptation became faster and more effective. We attribute changes in adaptation partly to a developmental switch from TMC2‐ to TMC1‐ containing channels and partly to an increase in channel expression. More complete and faster adaptation, coupled with larger MET currents, may account for the sole use of TMC1 in the adult cochlear hair cells.

## Key points

- Hair cell mechanoelectrical transducer channels are opened by deflections of the hair bundle about a resting position set by incompletely understood adaptation mechanisms.
- We used three characteristics to define adaptation in hair cell mutants of transmembrane channel‐like proteins, TMC1 and TMC2, which are considered to be channel constituents.
- The results obtained demonstrate that the three characteristics are not equivalent, and raise doubts about simple models in which intracellular Ca^2+^ regulates adaptation.
- Adaptation is faster and more effective in TMC1‐containing than in TMC2‐containing transducer channels. This result ties adaptation to the channel complex, and suggests that TMC1 is a better isoform for use in cochlear hair cells.
- We describe a TMC1 point mutation, D569N, that reduces the resting open probability and Ca^2+^ permeability of the transducer channels, comprising properties that may contribute to the deafness phenotype.

## Introduction

Sound stimuli are converted into electrical signals by the opening of mechanoelectrical transducer (MET) channels in the stereociliary bundle projecting from the top each hair cell. The MET channels are considered to be activated by tension in extracellular tip links connecting adjacent stereocilia along the bundle's excitatory axis (Assad *et al*. 1991; Beurg *et al*. 2009; Fettiplace & Kim, 2014). The maximum vibration of the stereociliary bundle at the loudest sound pressures is a fraction of a micron, less than the diameter of individual stereocilia, and so transduction is endowed with several processes of adaptation segregated into fast and slow phases (Eatock, 2000; Fettiplace & Kim, 2014). A role for adaptation is to ensure that MET channel activation always occurs around the ambient position of the bundle, thus maximizing sensitivity. The mechanisms of neither fast, nor slow adaptation are fully understood. Both were initially documented in non‐mammalian hair cells of frogs and turtles (Howard & Hudspeth, 1987; Crawford *et al*. 1989), where adaptation was considered to be driven by elevation of cytoplasmic Ca^2+^ that has entered via the MET channels (Eatock *et al*. 1987; Crawford *et al*. 1989; Hacohen *et al*. 1989). In such animals, adaptation is sensitive to the concentrations of extracellular Ca^2+^, intracellular calcium buffer, and the membrane potential driving Ca^2+^ entry (Ricci *et al* 1998). It has been proposed that fast adaptation is a result of Ca^2+^ interacting with the MET channels to adjust their sensitivity (Crawford *et al*. 1989; Cheung & Corey, 2006) and that, with slow adaptation, Ca^2+^ promotes slippage of the upper attachment point of the tip link, thereby reducing the mechanical stimulus to the channels (Howard & Hudspeth, 1987; Assad & Corey, 1992). However, there is little direct evidence for either process. Furthermore, the mechanisms are more uncertain in mammalian cochlear hair cells, where factors altering Ca^2+^ entry or internal Ca^2+^ buffering were reported to have little effect on either adaptation kinetics or steady‐state adaptation (Peng *et al*. 2013) and certain aspects of adaptation have been proposed to arise instead by extracellular Ca^2+^ modulating the membrane lipid (Peng *et al*. 2016). However, these conclusions have been controversial (Corns *et al*. 2014; Beurg *et al*. 2015) and two important questions persist: is Ca^2+^ necessary to trigger fast adaptation and does the cation interact directly with the MET channel? We have addressed these issues by characterizing fast adaptation and Ca^2+^ influx through the MET channel in mutations of transmembrane channel‐like (TMC) protein isoforms 1 and 2 (TMC1 and TMC2).

TMC1 and TMC2 play a pivotal role in MET channel function (Kurima *et al*. 2002; Kawashima *et al*. 2011). The two TMC isoforms have been localized to the site of the MET channels at the tips of the shorter stereocilia (Kurima *et al*. 2015) and a mutation in either isoform alters ion conduction through the MET channels (Kim & Fettiplace, 2013; Pan *et al*. 2013; Beurg *et al*. 2014). TMC2 is expressed early in mouse post‐natal development but is replaced by TMC1 by the end of the first postnatal week (Kawashima *et al*. 2011; Beurg *et al*. 2018). *Tmc1^–/–^* mice lack MET currents in OHCs after postnatal day (P)8 and are deaf (Kawashima *et al*. 2011; Kim & Fettiplace, 2013). We have also examined a *Tmc1* p.D569N mutation,(Kitajiri *et al*. 2007), a semi‐dominant mutation linked to progressive hearing loss.

## Methods

### Ethical approval

The care and use of animals for all experiments described conformed to NIH guidelines, and were approved by the Institutional Animal Care and Use Committees at the University of Wisconsin–Madison (approval reference M006211).

### Mouse mutants

The *Tmc1 *mutant mouse was B6.129‐*Tmc1^tm1.1Ajg^*/J (Kawashima *et al*. 2011) and was obtained from Jackson Labs (Bar Harbor, ME, USA; stock number 01 9146). The *Tmc2 *mutant mice (B6.129S5‐*Tmc2^tm1Lex^*/Mmucd) were obtained from the Mutant Mouse Regional Resource Centre (University of California, Davis, CA, USA). *Tmc1* p.D569N mice were made using a CRISPR technique by Applied StemCell Inc. (Milpitas, CA, USA) and the mutation was verified by 500 bp sequencing around the mutation site. *Tmc1 *p.M412K (*Beethoven*) mice were a gift from Karen Steel (Kings College London, London, UK). Both C57B6 and CD1 mice were used as controls. Neonatal mice were killed by decapitation in accordance with an animal protocol approved by the Institutional Animal Care and Use Committees at the University of Wisconsin–Madison. For all strains, a mixture of male and female mice was used and no gender‐specific effects were noted. Mice were kept under a 12:12 h light/dark photocycle and were allowed solid food and water *ad libitum*.

### Electrophysiology and stimulation

MET currents were recorded from outer hair cells (OHCs) and inner hair cells (IHCs) in isolated organs of Corti of mice between embryonic day 18 and postnatal day 10 (E18 to P10, where E19 = P0 is the birth day) using methods described previously (Kim & Fettiplace, 2013; Beurg *et al*. 2014). When documenting developmental changes, serial measurements were made on pups from a given litter at different stages of development, 24 h apart, and the results were averaged for three or more separate litters. Recording and stimulation methods were identical to those described previously (Kim *et al*. 2013; Beurg *et al*. 2014). Excised cochlear turns were immobilized in a recording chamber on a fixed‐stage microscope (DMFS; Leica Microsystems, Wetzlar, Germany) and viewed through a 63× long working distance water‐immersion objective. Apical and basal turns were ∼80% and ∼20%, respectively, of the distance along the cochlea from the stapes. The recording chamber was perfused with saline of composition (in mm): 152 NaCl, 6 KCl, 1.5 CaCl_2_, 2 Na‐pyruvate, 8 d‐glucose and 10 Na‐Hepes, pH 7.4, at 21–23 °C. Electrical recordings were made with borosilicate patch electrodes, filled with a solution (in mm): 128 CsCl, 3.5 mgCl_2_, 5 Na_2_ATP, 10 Tris phosphocreatine, 1,2‐bis‐(*O*‐amino‐phenoxy)‐ethane‐*N*,*N*,*N*',*N*'‐tetraacetic acid (BAPTA) and 10 Cs‐Hepes, pH 7.2, and connected to an Axopatch 200B amplifier (Molecular Devices, Sunnyvale, CA, USA). Voltage clamp protocols were usually referred to a holding potential of −84 mV. This potential represented a compromise: a larger holding potential increased the current amplitude and therefore improved the accuracy of the measurements, although, if it were too large (−100 mV), it shortened and destabilized the recordings. Because the MET current–voltage relationship is approximately linear, inferences about behaviour at the normal resting potential of −50 mV (Johnson *et al*. 2011) will be reasonably accurate. The potential across the MET channel in the hair bundle membrane *in vivo* is augmented by an endolymphatic potential that depends on age (Steel & Barkway, 1989). Uncompensated electrode series resistance was 5–10 MΩ, giving recording time constants of 25 to 50 µs. Experiments were performed at room temperature, 21–23 °C.

**Figure 1:**
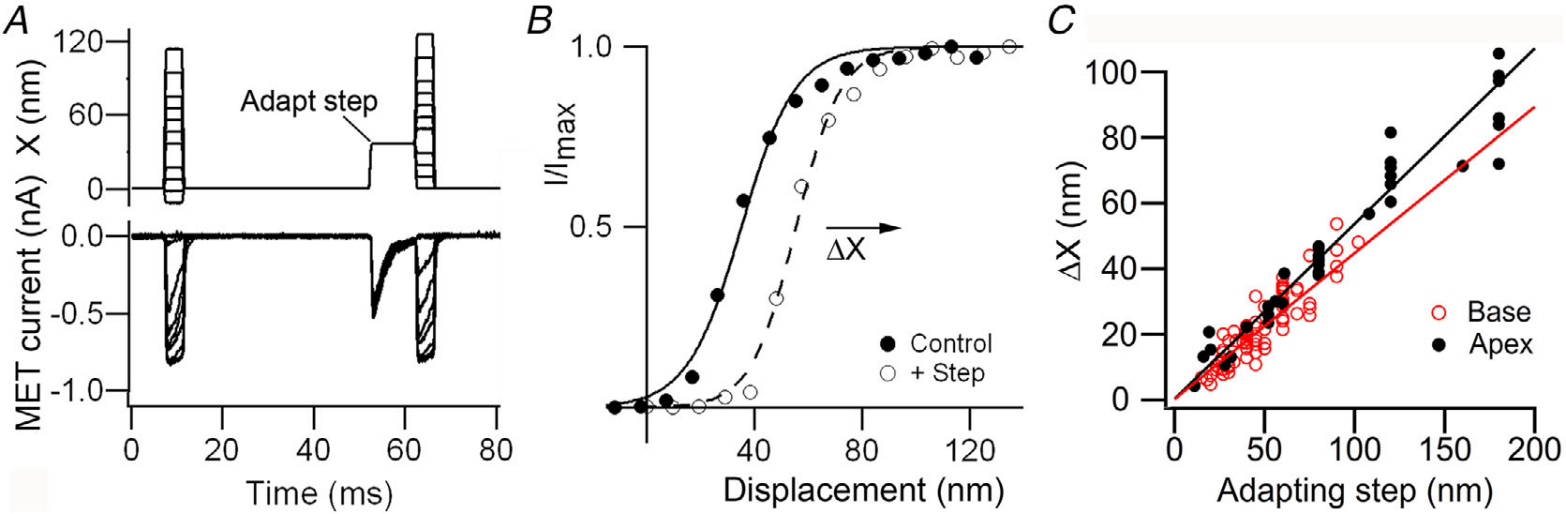
Adaptation assayed with two‐pulse experiment in a wild‐type OHCs *A*, MET currents in a P6 apical OHC for two series of 4 ms bundle displacements, the first control steps and the second test steps, with the test steps being preceded by a 10 ms adapting step. Note the current decay during the adapting step. *B*, current–displacement relationships for the OHC in (*A*) in response to the first (control) pulse and second (test) pulse during the adapting step. The current, *I*, is scaled to its maximum value, *I*_max_, 0.84 nA. Note the positive adaptive shift, Δ*X*, along the displacement axis. *C*, plot of shift in current–displacement relation, Δ*X*, *vs*. the size of adapting step for five P6 OHCs from apex (black circles), and five P3 and P4 OHCs from base (red circles). All currents measured at a holding potential of –84 mV. [Color figure can be viewed at http://wileyonlinelibrary.com]

Stereociliary bundles were stimulated with a fluid jet or a stiff glass probe driven by a piezoactuator and displacements of the bundle were calibrated by projection on a photodiode pair (Crawford & Fettiplace, 1985). A fluid jet stimulator caused less damage to the bundle and was able to push and pull equally, making is suitable for characterizing the level dependence of adaptation (Fig. 1). However, the stimulus onset was slower, and so the kinetics of adaptation were determined using a stiff glass probe driven by a piezoactuator (Kennedy *et al*. 2003); the driving voltage to the piezoactuator was filtered at 3 kHz, giving a stimulus rise time of ∼70 µs.

### Calcium selectivity

The Ca^2+^ permeability of the MET channel was determined (Beurg *et al*. 2006; Kim & Fettiplace, 2013) by measuring the Ca^2+^ reversal potential of the MET current in the presence of an intracellular CsCl‐based solution (composition in mm: 135 CsCl, 3 mgATP, 10 Tris phosphocreatine, 1 EGTA‐CsOH and 10 Hepes, pH 7.2) and an extracellular Ca^2+^ solution (composition in mm: 100 CaCl_2_, 20 *N*‐methylglucamine, 6 Tris and 10 d‐glucose, pH 7.4). The extracellular solution was applied by local perfusion and was also included in the fluid jet. Reversal potentials were corrected for the liquid junction potential of −9 mV, and the permeability of Ca^2+^ relative to Cs^−^, *P*_Ca_/*P*_Cs_, was calculated from the Goldman–Hodgkin–Katz equation using ion activity corrections as specified previously (Kim & Fettiplace, 2013).

### Statistical analysis

All results are reported as the mean ± SD unless otherwise stated. Statistical significance was investigated using a two‐tailed *t* test.

## Results

### TMC1 channels have stronger adaptation than TMC2 channels

**Figure 2:**
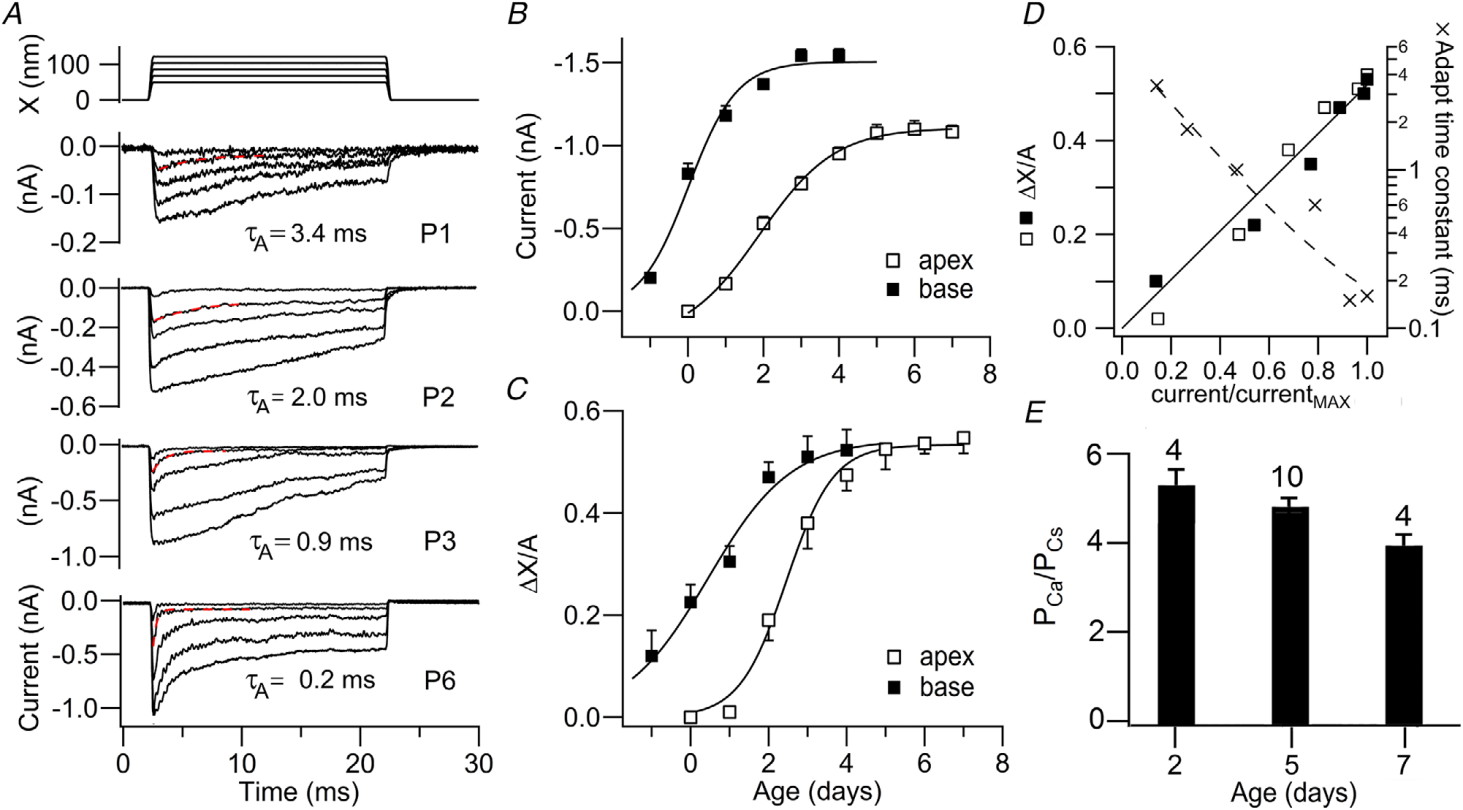
Postnatal development of fast adaptation in cochlear OHCs of wild‐type mice *A*, typical MET current families in apical OHCs in response to hair bundle deflection, *X*, with a stiff probe, recorded at different postnatal ages, P1 to P6, showing an increase in current amplitude. Time course of adaptive decline in small current responses fit with single exponential (dashed red line) with time constant τ_A_ given below. Currents measured at −84 mV holding potential. *B*, development of MET current magnitude in OHCs from cochlear apex (open squares) and base (filled squares). *C*, development of adaptation effectiveness, shift in activation curve, Δ*X*, produced by a given adapting step, *A*. *D*, Δ*X*/*A* results from (*C*) are proportional to MET current, scaled to its maximum value, from (*B*) in both apical (open squares) and basal OHCs (filled squares). Adaptation time constant from stiff‐probe recordings such as those in (*A*) decreases with current scaled to its maximum value (1.13 nA; crosses) for apex. *E*, Ca^2+^ permeability of the wild‐type MET channel of apical OHCs relative to Cs^+^ (*P*_Ca_/*P*_Cs_, mean ± SD, number of cells above each bar) decreases with developmental age, probably as a result of transition from TMC2‐containing to TMC1‐containing channels.

A distinctive property of MET channels is fast adaptation, which was characterized in OHCs from two‐pulse experiments (Beurg *et al*. 2015), and from the time course of adaptation for small displacements. In the two‐pulse experiment, the extent of adaptation was determined from the shift, Δ*X*, in the relationship between the MET current and the hair bundle displacement produced by a series of adapting steps, *A* (Fig. 1). The slope of the plot of Δ*X* against *A* provided a reproducible measure of the extent of adaptation over the linear range of stimulus amplitudes. In OHCs of wild‐type mice, the parameter, Δ*X/A*, increased over the first few postnatal days, to attain an asymptotic value, Δ*X/A* = 0.52; the change in adaptation at the base of the cochlea preceded that at the apex by ∼2.5 days, identical to the apex–base time difference in current magnitude (Fig. 2 *A–C*). The change in adaptation occurred in parallel with the growth of the maximum MET current and the two were correlated for both apex and base (Fig. 2 *D*). Accompanying the change in the extent of adaptation was a decrease in the adaptation time constant (Waguespack *et al*. 2007; Lelli *et al*. 2009), to a limit at P7 of 0.16 ± 0.03 ms (*n* = 6), as also plotted in Figure 2 *D* against the normalized MET current. Adaptation time constants were measured using a stiff probe and are plotted only for the apex. As noted previously in rats (Kennedy *et al*. 2003), at a given age, the adaptation time constant becomes faster with an increase in the maximum current, for which the bundle may contain more functional channels. Paradoxically, the development of fast adaptation over the first week occurred despite a 20% decrease in the Ca^2+^ permeability of the MET channel (Fig. 2 *E*).

**Figure 3:**
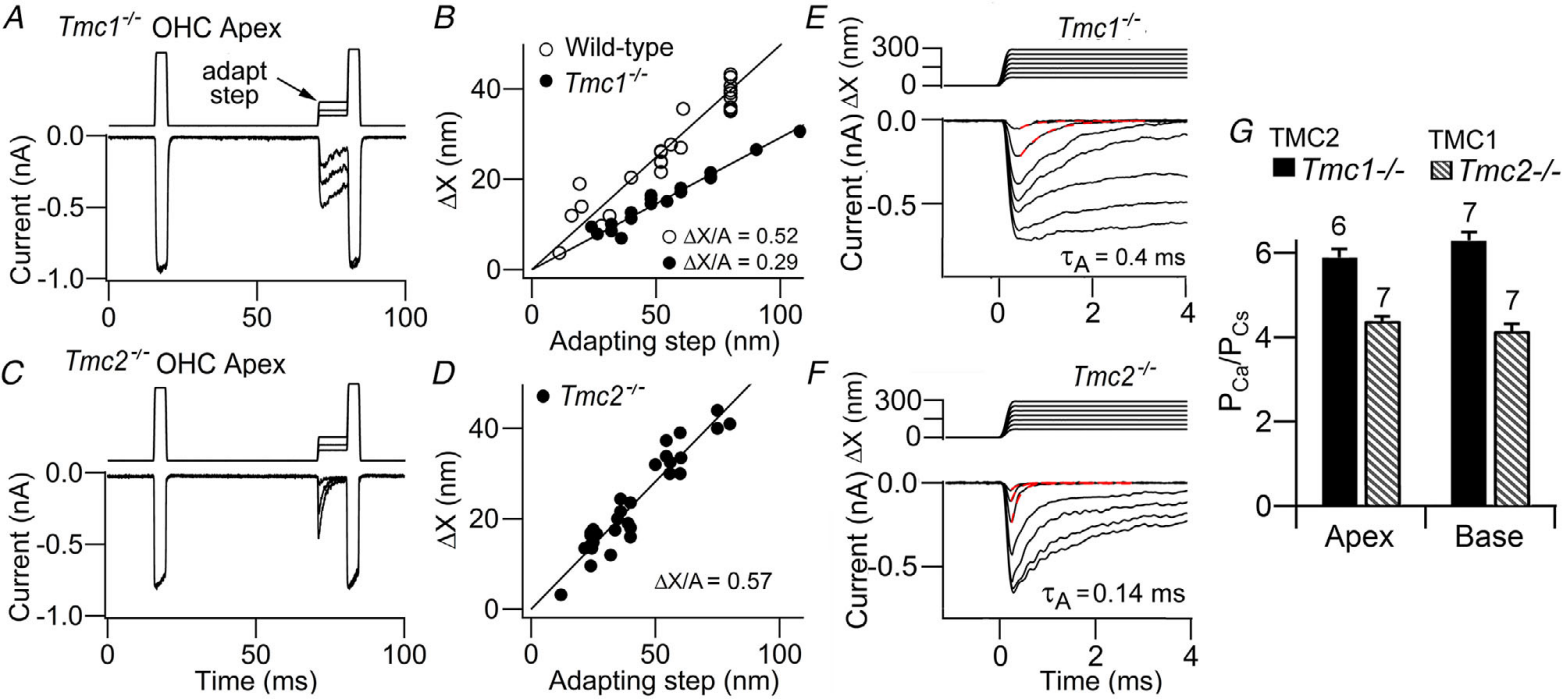
TMC1‐containing MET channels show faster and more complete adaptation than TMC2‐containing channels *A*, two‐pulse experiment with an initial control family of hair bundle displacement steps (only one out of 12 test steps is shown; see Fig. 1), then a second test family of displacement steps (only one out of 12 test steps is shown), with three levels of adapting step in OHC of *Tmc1^−/−^* mouse. *B*, shift in activation curve, Δ*X*, produced by a given adapting step, A, in five wild‐type and five *Tmc1^−/−^* mice. Δ*X*/*A* = 0.52 in wild‐type and 0.29 in *Tmc1^−/−^*. *C*, two‐pulse experiment (only one out of 12 test steps is shown) with second test displacement steps preceded by three adapting steps in OHC of *Tmc2^−/−^* mouse. *D*, shift in activation curve, Δ*X*, produced by a given adapting step, in six *Tmc2^−/−^* mice (ΔX/A = 0.57). *E*, MET current onsets for family of displacement steps in *Tmc1^−/−^*; red dashed lines, adaptive decline, time constant 0.4 ms. *F*, MET current onsets for family of displacement steps in *Tmc2* *^–/–^;* the red dashed lines, adaptive decline, time constant 0.14 ms; holding potential −84 mV. Note Δ*X*/*A* in TMC1‐channels (in *Tmc2^−/−^* mice) twice the value of Δ*X*/*A* in TMC2‐channels (in *Tmc1^−/−^* mice) and adaptation twice as fast. *G*, Ca^2+^ permeability of the MET channel relative to Cs^+^ (*P*_Ca_/*P*_Cs,_ mean ± SD, seven OHCs for each measurement) for *Tmc2^−/−^* and *Tmc1^−/−^* mice at cochlear apex and base. Mouse postnatal ages: P4 (*A*); wild‐type, P6–P7; *Tmc1^−/−^*, P3–P5 (*B*); P6 (*C*); P6–P7 (*D*); P5 (*E*); P7 (*F*); P3–P6 (*G*).

There are changes in the expression of TMC2 and TMC1 in the first few postnatal days. TMC2‐dependent MET channels first occur but, after a few days, they are replaced by TMC1‐dependent channels (Beurg *et al*. 2018). We therefore characterized adaptation in the *Tmc1^–/–^* and *Tmc2^–/–^* mice to determine whether differences in adaptation might arise as a result of different TMC isoforms (Fig. 3 *C–F*). In six OHCs of *Tmc1^–/–^* mice, the extent of adaptation was significantly reduced (Δ*X /A* = 0.29 ± 0.01; mean ± SEM) and was one‐half the value of that in the *Tmc2^–/–^* mice (Δ*X /A = *0.57 ± 0.01; mean ± SEM). The difference is significant (two‐tailed *t* test, *p* < 0.001), despite there being little difference in MET current amplitudes between *Tmc1^–/–^* OHCs (0.88 ± 0.11 nA, *n* = 6) and *Tmc2^–/–^* OHCs (0.81 ± 0.01 nA, *n* = 4). Although it was clear that adaptation was also faster in the *Tmc2^–/–^* than in *Tmc1^–/–^* (Fig. 3 *A* and *C*), the use of the slower fluid jet stimulation underestimates the speed, and so we measured the adaptation kinetics with a stiff probe (Fig. 3 *E* and *F*). In *Tmc2^–/–^* mice, the mean adaptation time constant, τ_A_, = 0.16 ± 0.08 ms (*n* = 7), whereas, in *Tmc1^–/–^* mice, τ_A_ = 0.41 ± 0.08 ms (*N* = 4), the two values were significantly different (*t* test, *p* < 0.001). The results indicate that adaptation is faster when the MET channel contains TMC1 alone (in the *Tmc2^–/–^*), rather than TMC2 (in the *Tmc1^–/–^*). Differential expression of the two isoforms may therefore partly explain the changes in the MET current size and adaptation that occur during development. However, even in the absence of TMC2, the MET current amplitude and fast adaptation still took more than 2 days to plateau, indicating that development is limited by another time‐dependent process. Although not extensively investigated, similar differential effects were seen in IHCs. Δ*X/A* was 0.29 ± 0.01 (*N* = 4) in *Tmc1^+/+^;Tmc2^+/+^* mice and Δ*X/A* was 0.40 ± 0.01 (*n* = 5) in *Tmc1^+/+^;Tmc2^–/–^* mice; maximum MET currents were 0.97 ± 0.03 nA in *Tmc1^+/+^;Tmc2^+/+^* and 0.55 ± 0.03 nA in *Tmc1^+/+^;Tmc2^–/–^*, both in P6 mice.The smaller current in *Tmc2^–/–^* at this age reflects the fact that TMC2 contributes significantly to the IHC MET channels (Beurg *et al*. 2018).

If adaptation is regulated by intracellular Ca^2+^ (Fettiplace & Kim, 2014), the increase in efficacy and speed could in theory stem from an increase in Ca^2+^ influx as a result of changes in channel current size or permeability to Ca^2+^. TMC2‐containing channels have a 17% smaller unitary conductance, 58 pS compared to 70 pS (Beurg *et al*. 2018), although this is offset by a 40% larger permeability to Ca^2+^ (*P*_Ca_/*P*_Cs_ = 6.0 for TMC2 compared to 4.2 for TMC1 (Kim & Fettiplace, 2013) (Fig. 3 *G*). The combination of these two parameters is too small to produce the much slower adaptation in TMC2‐containing channels, and some other difference in the channel or its environment must be identified. A correlation between the speed of adaptation and current size has been demonstrated previously (Ricci & Fettiplace, 1997; Kennedy *et al*. 2003) and it is possible that this contributes to the changes in adaptation during development. However, the simplest conclusion is that the adaptation not only depends on the Ca^2+^ influx, but also on the composition of the MET channel, whether containing TMC1 or TMC2. For either TMC1 or TMC2, there was no significant difference in Ca^2+^ permeability between apex and base (Fig. 3 *G*).

### *Tmc1* p.D569N mutation

**Figure 4:**
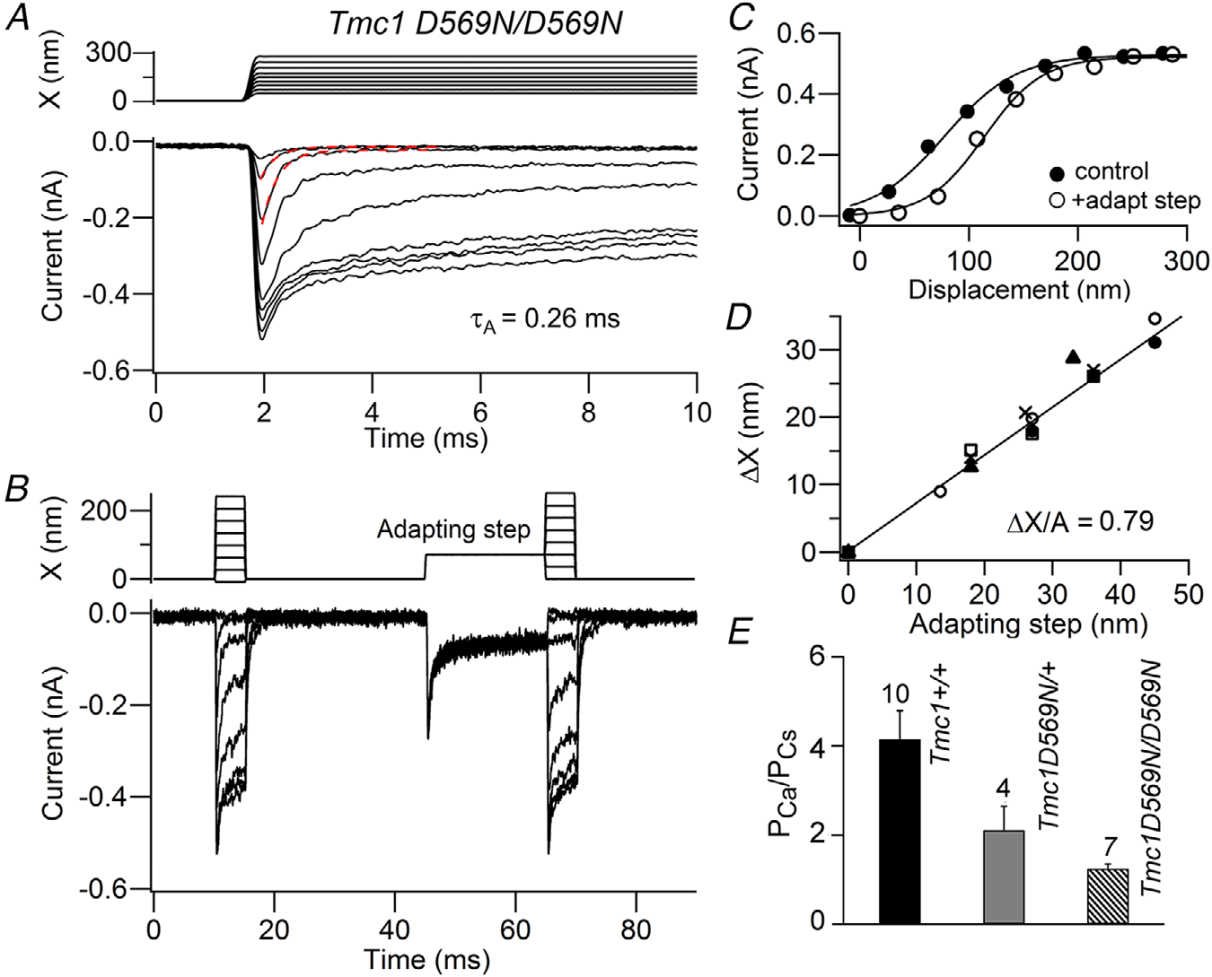
Adaptation in OHCs from *Tmc1* p.D569N/D569N*;Tmc2* *^−/−^* mice *A*, MET current onsets for family of hair bundle displacement steps; red dashed lines are fits to adaptive decline with time constant 0.26 ms; measurements with glass probe stimulator in a P7 apical OHC at –84 mV holding potential (*B*), two‐pulse experiment with an initial control family of hair bundle displacement steps, then a second test family of displacement steps preceded by adapting steps of different magnitude. *C*, current–displacement relationships from record in (*B*), for first pulse (filled symbols) and second pulse (open symbols) showing shift in activation curve, Δ*X*, produced by the adapting step, each fit with single Boltzmann equation. *D*, plot of shift in activation curve, Δ*X*, produced by adapting steps in P7 apical OHCs of five *Tmc1 p.D569N* mice, slope Δ*X*/*A* = 0.79. *E*, reversal potential and MET channel permeability of Ca^2+^ relative to Cs^−^ (*P*_Ca_/*P*_Cs_, mean ± SD, number of cells above each bar) for *Tmc1^+/+^;Tmc2^−/−^*, *Tmc1* p.D569N/+*;Tmc2^−/−^* and *Tmc1* p.D569N/D569N*;Tmc2^–/–^*.

The relationship between TMC1 and adaptation was also addressed using a *Tmc1 *mutant containing a single amino acid replacement, D569N (aspartate569 being replaced by asparagine). The equivalent human mutant is dominant and linked to progressive hearing loss (Kurima *et al*. 2002; Kitajiri *et al*. 2007). We have shown that, from acoustic brainstem responses, both homozygotes and heterozygote *Tmc1* p.D569N mice were completely deaf by P30 (Beurg *et al*. 2019). However, early in neonatal development, MET currents were recordable from OHCs at P6 in *Tmc1* p.D569N mice and such currents were not attributable to TMC2 because they were present in *Tmc2^–/–^*. MET currents in *Tmc1* p.D569N mutant mice were smaller than those in *Tmc1^+/+^* but nevertheless displayed fast adaptation, with a mean time constant of 0.23 ± 0.04 ms (*n* = 6) in OHCs and with maximum currents of 0.4–0.5 nA (Fig. 4 *A*). Mutant MET channels also showed an adaptive shift in the transducer activation curve (Fig. 4 *B* and *C*). A plot of the shift in the activation curve, Δ*X*, against the adapting step, *A*, (Fig. 4D) had a slope Δ*X/A* of 0.79 ± 0.06 (*n* = 5), indicating that the MET channels possess adaption as effective as, if not better than, the control channels in OHCs of *Tmc1^+/+^* mice. Statistical tests on the Δ*X/A* showed a significant difference to control (*t* test, *p* < 0.001), although there was no significant difference in the fast adaptation time constant between the two strains. Enhanced adaptation in *Tmc1* p.D569N might be attributable to an increased Ca^2+^ permeability for the MET channel. However, measurements of reversal potentials for the MET current showed (Fig. 4 *E*) that, in contrast, there was a substantial reduction in *P*_Ca_/*P*_Cs_ for the *Tmc1* p.D569N, from *P*_Ca_/*P*_Cs_ = 4.20 ± 0.7 (*n* = 5) for the *Tmc1^+/+^* control mice to *P*_Ca_/*P*_Cs_ = 1.24 ± 0.1 (*n* = 7) for the *Tmc1* p.D569N/D569N homozygote, and *P*_Ca_/*P*_Cs_ = 2.11 ± 0.5 (*n* = 6) for the *Tmc1* p.D569N/+ heterozygote. The heterozygote and homozygote are significantly different (*p* = 0.01), as are the heterozygote and control (*t* test, *p = *0.004).

### Resting probability of MET channel opening

**Figure 5:**
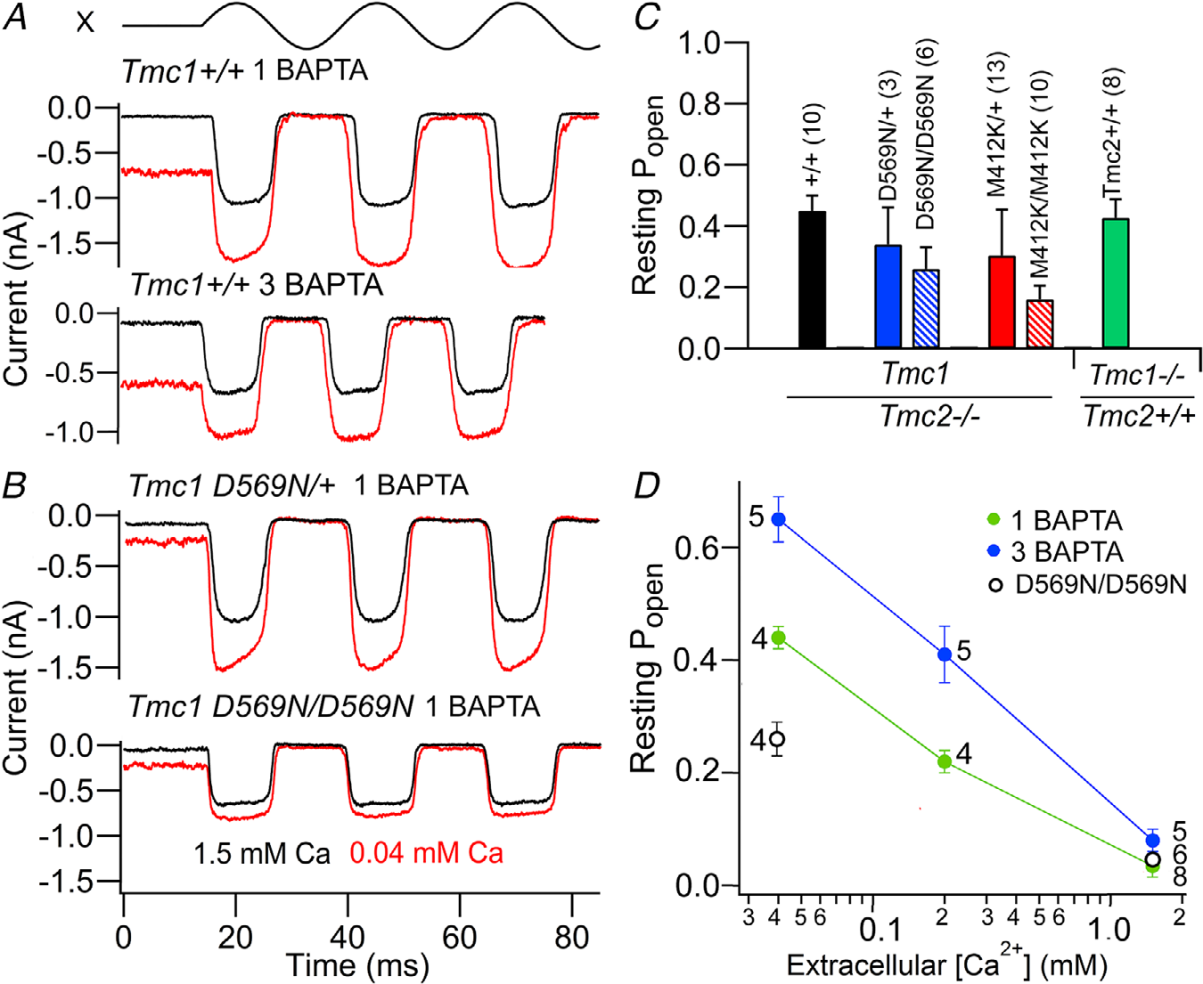
Effects of extracellular Ca^2+^ and cytoplasmic Ca^2+^ buffer on MET channel resting open probability *A*, receptor currents for sinusoidal deflections of OHC hair bundles in 1.5 mm external Ca^2+^ (black traces) and 0.04 mm external Ca^2+^ (red traces). Two different recordings: *Tmc1^+/+^* with 1 mm intracellular BAPTA; *Tmc1^+/+^* with 3 mm intracellular BAPTA. *B*, *Tmc1* p.D569N/+ with 1 mm intracellular BAPTA; *Tmc1* p.D569N/D569N with 1 mm intracellular BAPTA. All measurements were conducted with a fluid jet stimulator in apical OHCs, −84 mV holding potential, P7 cochlea apex. *C*, mean ± SD of resting open probability, *P*_OR_, of MET channels in 0.04 mm external Ca^2+^ and 1 mm internal BAPTA in the following mutants: *Tmc1^+/+^; Tmc2^−/−^*; *Tmc1* p.D569N/+; *Tmc2^−/−^*; *Tmc1* p.D569N/D569N*;Tmc2^−/−^; Tmc1* p.M412K/*+;Tmc2^−/−^; Tmc1* p.M412K/M412K*;Tmc2^−/−^; Tmc1^−/−^;Tmc2^+/+^* *. Tmc1* p.M412K is *Beethoven*, another semi‐dominant mutant. Note *P*_OR_ is reduced in both *Tmc1 *mutants, the effects being larger in the heterozygote than in the homozygote. *D*, resting open probability of MET channels as a function of external Ca^2+^ and intracellular BAPTA concentration in *Tmc1^+/+^* (green and blue points) and in *Tmc1* p.D569N/D569N (white points).

An important functional attribute of the MET channel in OHCs, related to adaptation, is the resting open probability (*P*_OR_) of the channel, reflecting the position of the activation curve along the displacement axis. When the hair bundles are bathed in saline containing low, 40 µm, Ca^2+^ similar to endolymph *in vivo* (Bosher & Warren, 1978; Ikeda *et al*. 1987), *P*_OR_ is 0.4–0.5 (Beurg *et al*. 2010; Johnson *et al*. 2011), compared to 0.03 when bathed in saline containing 1.5 mm Ca^2+^ such as perilymph. Thus, lowering extracellular Ca^2+^ substantially increases *P*_OR_ (Fig. 5 *A*), which serves to generate a substantial depolarizing inward current flowing via partially open MET channels (Johnson *et al*. 2011). An important functional consequence is that the large depolarizing current offsets an equivalently large outward current flowing through voltage‐dependent K^+^ channels in the basolateral membrane; together, these two currents result in an OHC resting potential of around –50 mV, near the steepest slope of the prestin activation curve, thus optimizing OHC amplification (Johnson *et al*. 2011). The increase in *P*_OR_ has been found to depend on the concentration of the cytoplasmic calcium buffer (Beurg *et al*. 2010; Johnson *et al*. 2011; Corns *et al*. 2014) (Fig. 5). With respect to the hypothesis that adaptation is regulated by cytoplasmic Ca^2+^, larger amounts of calcium buffer reduce the Ca^2+^ concentration at the cytoplasmic face of the MET channel and lead to channel opening. In line with this hypothesis, *P*_OR_ increases both on lowering the extracellular [Ca^2+^] and on elevating the concentration of the intracellular calcium buffer, BAPTA (Fig. 5 *D*).

There was a significant reduction in *P*_OR_ for *Tmc1* p.D569N homozygotes and an intermediate effect was seen with heterozygote compared to the homozygote (Fig. 5 *B*). For comparison, values for the *Tmc1* p.M412K mutation are also shown (Beurg *et al*. 2015; Corns *et al*. 2016). All measurements with the mutants were conducted using 1 mm BAPTA in the cytoplasmic solution. These effects with the mutants are paradoxical compared to the direct consequences of lowering extracellular Ca^2+^ or elevating cytoplasmic calcium buffering. Thus, if the Ca^2+^ influx is reduced by lowering extracellular Ca^2+^, then *P*_OR_ increases, whereas, if Ca^2+^ influx is reduced by lowering the MET channel Ca^2+^ permeability, then, in this mutant, *P*_OR_ decreases. However, this would be consistent with an increased adaptation in the mutant (Fig. 4). Despite this result with *Tmc1* p.D569N, the effects of external Ca^2+^ block were identical to wild‐type, with the maximum current increasing by the same amount on lowering extracellular Ca^2+^ from 1.5 to 0.04 mm. Thus, the ratio of OHC MET current in 0.04 mm Ca^2+^ to that in 1.5 mm Ca^2+^ was 1.46 ± 0.2 (*n* = 9) in *Tmc1^+/+^*, whereas the ratio was 1.58 ± 0.1 (*n* = 3) in *Tmc1* p.D569N. There was no difference between the ratios in the control and mutant (*t* test, *p = *0.53). This result suggests that D569 is not the site at which Ca^2+^ blocks the MET current (Fettiplace & Kim, 2014), even though that residue may contribute to Ca^2+^ permeability.

### Control of adaptation by extracellular Ca^2+^

**Figure 6:**
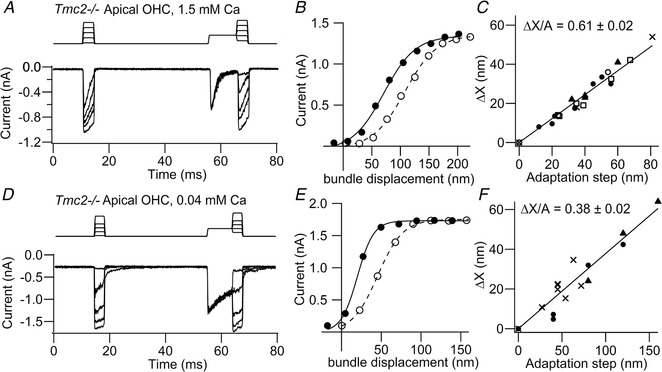
Effects of external Ca^2+^ on adaptation in apical OHCs from *Tmc1^+/+^;Tmc2* *^−/−^* mice *A*, two‐pulse experiments (only one adapting step shown) in saline containing 1.5 mm external Ca^2+^. *B*, Current–displacement relationships for first (filled circles) and second (open circles) displacement steps in (*A*) showing adaptive shift in curve, Δ*X*. *C*, Δ*X* plotted against adapting step, *A*, for five OHCs, slope Δ*X*/*A* = 0.61 ± 0.02. *D*, two‐pulse experiments (only one adapting step shown) in saline containing 0.04 mm external Ca^2+^. *E*, current–displacement relationships for first (filled circles) and second (open circles) displacement steps in (*D*) showing adaptive shift in curve, Δ*X*. *F*, Δ*X* plotted against adapting step, *A*, for three OHCs, slope Δ*X*/*A* = 0.38 ± 0.02. All measurements were performed at −84 mV holding the potential with a fluid jet stimulator.

With respect to the hypothesis that Ca^2+^ is involved in regulating adaptation, the properties of adaptation should be a function of external Ca^2+^. These were examined in OHCs of *Tmc1^+/+^;Tmc2^–/–^* mice using the fluid jet stimulator and a two pulse experiment (Fig. 6). Control measurements in 1.5 mm Ca^2+^ gave a mean Δ*X/A* of 0.61 ± 0.02 (*n* = 5 OHCs). On reducing the extracellular Ca^2+^ around the hair bundle to 40 µm, the MET current and resting open probability increased but Δ*X/A* showed a significant decrease to 0.38 ± 0.02 (*n* = 3 OHCs) (*t* test, *p* < 0.001). Initially, these results appear to imply that reducing Ca^2+^ influx, either by lowering external Ca^2+^ concentration or reducing the MET channel Ca^2+^ permeability should diminish adaptation. However, the dependency of Δ*X/A* on external Ca^2+^ is a necessary but not sufficient condition to conclude that adaptation is controlled by cytoplasmic Ca^2+^, and it is possible that the regulation occurs at the external face of the channel.

**Figure 7:**
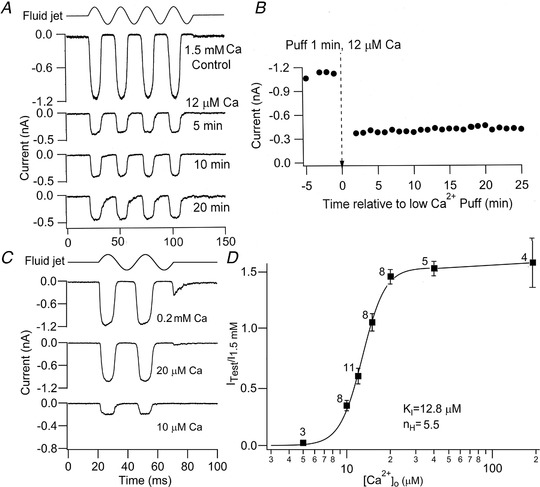
Effects of external Ca^2+^ maintenance of MET current in apical OHCs from wild‐type mice *A*, control measurement in 1.5 mm Ca^2+^ followed by exposure to 12 µm Ca^2+^ for time shown. *B*, MET current amplitude is stable after switching to 12 µm Ca^2+^. *C*, examples of MET current after switching from 1.5 mm Ca^2+^ to tests of 0.2 mm, 20 µm and 10 µm Ca^2+^. *D*, plot of *I*_Test_/*I*_1.5 mm_ *vs*. test external Ca^2+^, mean ± SEM, number of OHCs measured shown above each point. Results fit with Hill equation with *K*_I_ = 12.8 µm and Hill coefficient *n*_H_ = 5.5.

External Ca^2+^ is known also be involved in the stability of the tip link, uniting the cadherin‐23 and protocadherin‐15 components (Kazmierczak *et al*. 2007). The dependence of the tip‐link integrity, manifested as channel activation, was investigated as a function of external Ca^2+^ concentration.The experiment was performed by obtaining a control MET current in saline containing 1.5 mm Ca^2+^, and then perfusing a low‐Ca^2+^ saline (Fig. 7 *A* and *B*) across the hair bundles. The current changed, taking ∼1–3 min to stabilize, although it then remained constant over 20 min or more. At the concentration of 40 µm Ca^2+^ used in the experiments described above, the MET current increased ∼50% above that in 1.5 mm Ca^2+^, reflecting unblocking of the channel (Fettiplace & Kim, 2014). If the Ca^2+^ was further reduced, the current declined irreversibly, most probably as a result of severance of the tip links (Fig. 7 *C* and *D*). Fitting the relationship between MET current and Ca^2+^ with the Hill equation gave a half‐inhibitory concentration, *K*_I_, of 12.8 µm; *n*_H_, the Hill coefficient of binding was 5.5, which may be taken as a lower limit on the number of Ca^2+^ ions involved in the interaction between two cadherin‐23 and two protocadherin‐15 filaments. Surprisingly, *K*_I_ is scarcely smaller than the purported Ca^2+^ concentration of 20 µm in endolymph (Bosher & Warren, 1978; Ikeda *et al*. 1987; Wood *et al*. 2004).

Tip‐link integrity is crucially dependent on external free Ca^2+^. However, the exact Ca^2+^ level near the tips of the stereocilia *in vivo* may be higher than that in the bulk endolymph, partly because of vigorous Ca^2+^ extrusion by the plasma membrane CaATPase, coupled with a diffusion barrier imposed by the tectorial membrane (Yamoah *et al*. 1998; Strimbu *et al*. 2019). Because the resting open probability is a function of endolymph Ca^2+^, *P*_OR_ may be used to estimate the free Ca^2+^ *in vivo*. *P*_OR_, sometimes referred to as the operating point of transduction, has been inferred in intact preparations from intracellular recordings to be ∼0.26 (Dallos, 1986), and from microphonic measurements to be ∼0.45 (Sirjani *et al*. 2004). Taking this range of values for *P*_OR_ and assuming the cytoplasmic calcium buffer is equivalent to 1 mm BAPTA (Johnson *et al*. 2011), from Figure 5D, the Ca^2+^ near the MET channel is in the range 40–150 µm. In the experiments shown in Figure 5, there was good mixing as a result of the fluid jet stimulator, and no diffusion barriers, and so the Ca^2+^ near the stereocilia is probably the same as that in the bulk solution.

## Discussion

We have characterized the fast adaptation of hair cell MET channels containing different TMC proteins, which are considered to be a molecular component of the channel (Kawashima *et al*. 2011; Pan *et al*. 2013; Fettiplace & Kim, 2014; Pan *et al*. 2018). Our results indicate that both the extent and time constant of adaptation depend on which TMC isoform is present, with the observations effectively localizing the adaptation mechanism to the channel complex. In particular, adaptation was faster and more complete in TMC1‐containing than in TMC2‐containing channels. However, if adaptation is regulated by Ca^2+^ influx, this disparity cannot be accounted for by differences in Ca^2+^ permeability between the two isoforms because TMC2 has the larger Ca^2+^ permeability with comparable unitary conductance. A mutation harboring a single amino acid replacement, D569N, in TMC1 had MET channels showing adaptation of comparable or greater extent compared to wild‐type TMC1 channels, although with substantially smaller Ca^2+^ permeability. According to prevailing models of TMC1, the D569 residue lies near the inner end of the hypothetical ion‐conducting pore of the channel (Ballesteros *et al*. 2018; Pan *et al*. 2018), which might account for the effect on Ca^2+^ permeability. These conflicting experimental findings raise concerns about the role of Ca^2+^ entry in adaptation, echoing previous conclusions (Peng *et al*. 2013; Peng *et al*. 2016).

We have used three processes to characterize fast adaptation: the extent, Δ*X/A*, inferred from two‐step protocols; the fast time constant of current decline, τ_A_; and the resting open probability, *P*_OR_, in 40 µm Ca^2+^. Our results show that these three properties do not lead to equivalent conclusions. For example, with TMC2, Δ*X/A* is smaller than with TMC1, consistent with a slower τ_A_, although *P*_OR_ is almost identical to TMC1. Another paradox is that *P*_OR_ is reduced in the *Tmc1* p.D569N mutant, suggesting a stronger adaptation, even though the Ca^2+^ permeability is smaller. The smaller *P*_OR_, by reducing the resting depolarizing current, is predicted to hyperpolarize OHCs and consequently diminish prestin‐based amplification (Johnson *et al*. 2011). This effect may be a significant factor contributing to the deafness phenotype. A similar behaviour was seen with the *Tmc1 *p.M412K (*Beethoven*) mutation (Beurg *et al*. 2015; Corns *et al*. 2016). A problem with respect to the underlying mechanism specifying *P*_OR_ is that it may be modulated by other processes (Peng *et al*. 2016). In many hair cell preparations, *P*_OR_ varies with concentrations of extracellular Ca^2+^ and intracellular calcium buffer BAPTA (Ricci *et al*. 1998; Beurg *et al*. 2010), as also reported in the present study for mouse cochlear OHCs (Fig. 5). These observations have been used to support the notion that the signal regulating adaptation is the stereociliary Ca^2+^ concentration. However, although BAPTA is a fast Ca^2+^ chelator widely used to study the roles of Ca^2+^ in cellular processes, there is evidence that it has other Ca^2+^ independent effects, including depolymerization of actin filaments and microtubules and depletion of ATP (Saoudi *et al*. 2004). Whether these effects are significant in the stereocilia remains unknown.

The fastest adaptation time constant, determined for TMC1‐containing MET channels, was 0.16 ms, with this time constant being measured for small bundle deflections that gave approximately linear responses. However, the time course of adaptation depends on the size of the stimulus and, for large displacements, it becomes progressively slower (Crawford *et al*. 1989), a behaviour exemplified in the P6 MET currents in Figure 2 *A*. A possible explanation for the prolongation in time course is that it is partly a result of saturation of the fast component with level, and partly a result of the appearance of another component of adaptation having a 10‐fold slower time constant (Wu *et al*. 1999; Vollrath & Eatock, 2003).The fast adaptation time constant is also temperature sensitive, as might be expected if it were limited by MET channel kinetics; a *Q*_10_ of between 2 and 3 has been measured in turtle auditory hair cells (Crawford *et al*. 1991). We previously extrapolated the kinetics of adaptation in mammalian OHCs from *in vitro* recordings at 22 °C to those expected in the mammalian cochlea *in vivo*; correction to body temperature (37 °C) and for the presence of an endolymphatic potential that summed with the resting potential predicted an eight‐fold shortening of the time constant (Kennedy *et al*. 2003; Ricci *et al*. 2005).

The differential expression of the TMC1 and TMC2 isoforms may partly explain the changes in the MET current adaptation that occur during development, with adaptation being improved by replacement of TMC2 with TMC1. However, even in the absence of TMC2, both current amplitude and fast adaptation still increase over 2 days, implying that development is limited by another time‐dependent process. Possibilities include transport of the TMC proteins up the stereocilia and their incorporation into a multimolecular transduction complex at the stereociliary tip. The changes in adaptation closely follow the increase in current size, a correlation between the two parameters having been demonstrated previously in turtles and rats (Ricci & Fettiplace, 1997; Kennedy *et al*. 2003). It is conceivable that this correlation reflects the fact that larger currents are attributable to greater numbers of channels at the transduction complex (Beurg *et al*. 2018). Our experiments indicate that the presence of TMC1 may be superior to TMC2 for optimizing the MET channel for OHC transduction. TMC1 provides a larger current and faster adaptation than required in vestibular hair cells, which are considered to retain TMC2 through adulthood (Kawashima *et al*. 2011; Kurima *et al*. 2015). It also endows an apical–basal gradient in unitary conductance, which, together with an increase in the numbers of stereocilia per bundle, augments the hair cell MET current several fold, thus enhancing sensitivity in basal high‐frequency OHCs.

TMC2 is a slightly larger protein than TMC1 (888 compared to 757 amino acids in mouse), with a longer N‐terminal region prior to the first transmembrane domain and C‐terminal region after the last transmembrane domain, both of which might be interfaces with other channel constituents. The N‐terminus is of particular interest because it is the region (residues 81–130 in TMC1) necessary for interaction with the Ca^2+^‐binding protein, CIB2 (Giese *et al*. 2017), which could theoretically mediate adaptation. The nearest family member to CIB2, CIB1, which is 59% similar, is an inhibitor of ion flux through the inositol triphosphate receptor Ca^2+^ channel (White *et al*. 2006; Hennigs *et al*. 2008).The N‐terminal interaction region of TMC1 is 80% similar to TMC2, which also interacts with CIB2 (Giese *et al*. 2017). Differences in fast adaptation between TMC1 and TMC2 might reflect the small differences in this interaction zone. Adaptation has been controversial for some time, although more work is still needed to fully clarify its origin.

## Additional information

### Competing information

The authors declare that they have no competing interests.

### Author contributions

ACG, MB and RF were responsible for the conception and design of the experiments. ACG and MB were responsible for the collection of the data. ACG, MB and RF were responsible for the analysis and interpretation of the data. ACG, MB and RF were responsible for drafting the article and revising it critically for important intellectual content. All authors approved the final version of the manuscript. All experiments were carried out at the Department of Neuroscience, University of Wisconsin–Madison, USA.

### Funding

The work was funded by grants RO1 DC015439 and RO1 DC01362 from the National Institute on Deafness and other Communication Disorders to RF.
